# The diffuse gamma-ray flux from clusters of galaxies

**DOI:** 10.1038/s41467-023-38226-w

**Published:** 2023-04-29

**Authors:** Saqib Hussain, Rafael Alves Batista, Elisabete M. de Gouveia Dal Pino, Klaus Dolag

**Affiliations:** 1 Institute of Astronomy, Geophysics and Atmospheric Sciences (IAG), University of São Paulo (USP), R. do Matão, 1226, 05508-090 São Paulo, Brazil; 2 Gran Sasso Science Institute, Via Michele Iacobucci, 2, 67100 L’Aquila, Italy; 3 Instituto de Física Teórica UAM-CSIC, C/ Nicolás Cabrera 13-15, 28049 Madrid, Spain; 4 Departamento de Física Teórica, Universidad Autónoma de Madrid, M-15, 28049 Madrid, Spain; 5 University Observatory Munich, Scheinerstr. 1, 81679 München, Germany; 6 Max Planck Institute for Astrophysics, Karl-Schwarzschild-Str 1, 85741 Garching, Germany

**Keywords:** Galaxies and clusters, Particle astrophysics, High-energy astrophysics

## Abstract

The origin of the diffuse gamma-ray background (DGRB), the one that remains after subtracting all individual sources from observed gamma-ray sky, is unknown. The DGRB possibly encompasses contributions from different source populations such as star-forming galaxies, starburst galaxies, active galactic nuclei, gamma-ray bursts, or galaxy clusters. Here, we combine cosmological magnetohydrodynamical simulations of clusters of galaxies with the propagation of cosmic rays (CRs) using Monte Carlo simulations, in the redshift range *z* ≤ 5.0, and show that the integrated gamma-ray flux from clusters can contribute up to 100% of the DGRB flux observed by Fermi-LAT above 100 GeV, for CRs spectral indices *α* = 1.5 − 2.5 and energy cutoffs ${E}_{\max }=1{0}^{16}-1{0}^{17}$ eV. The flux is dominated by clusters with masses 10^13^ ≲ *M*/*M*_⊙_ ≲ 10^15^ and redshift *z* ≲ 0.3. Our results also predict the potential observation of high-energy gamma rays from clusters by experiments like the High Altitude Water Cherenkov (HAWC), the Large High Altitude Air Shower Observatory (LHAASO), and potentially the upcoming Cherenkov Telescope Array (CTA).

## Abstract (web summary)

The origin of the diffuse gamma-ray background (DGRB) is unknown. Here, the authors show that the integrated gamma-ray flux from clusters can contribute up to 100% of the DGRB flux observed by Fermi-LAT above 100 GeV.

## Introduction

The DGRB provides a unique glimpse into the high-energy universe. Its inherent links with high-energy CRs and neutrinos enable investigations of the most powerful cosmic accelerators in the Cosmos. The observed energy fluxes of these three components are all comparable^1–3^, suggesting that they may have a common origin. Galaxy clusters are believed to be the result of very violent processes such as the accretion and merging of smaller structures into larger ones. These processes can release large amounts of energy (about 10^60^ − 10^64^ erg), part of which can accelerate CRs to very-high energies^4–6^. CRs with *E* ≲ 10^17^ eV can be confined within clusters for a time comparable to the age of the universe due to the size of these structures (of the order of Mpc) and their magnetic-field strength (*B* ~ *μ*G)^4,7^. Therefore, clusters are unique reservoirs of CRs that can produce high-energy photons through collisions with the gas in intracluster medium (ICM), or through processes involving energetic electron-positron pairs produced as secondaries of hadronic and/or leptonic interactions. CR interactions with the cosmic microwave background (CMB) and the extragalactic background light (EBL) are also promising channels for producing high-energy gamma rays, especially for CRs with energies ≳10^18^ eV.

Several analytical and semi-analytical models have been employed to estimate the fluxes of gamma rays and neutrinos stemming from CR interactions in the ICM^2,8–12^, but in all these studies the ICM is assumed to have spherically symmetric distributions of magnetic fields and gas.

Here, we explore the production of DGRB by galaxy clusters. We adopt a more rigorous numerical approach, employing cosmological three-dimensional magnetohydrodynamic (3D-MHD) simulations^7^, taking into account the non-uniform distributions of the gas density, temperature, and magnetic field, as well as their dependence on the mass and redshift of the clusters. We did not make any approximations to constrain the background density, temperature, and magnetic fields of the ICM as they are directly obtained from the simulations. This extends our previous work in which we employed a similar approach to compute the diffuse neutrino emission from these structures^13^. Our cosmological simulations indicate that the magnetic field and gas density distributions in massive clusters (with *M* ≳ 10^14^ *M*_⊙_) are larger than in the lower-mass ones, and that massive clusters (*M* ≳ 10^15^ *M*_⊙_) are less abundant at high redshifts^13–16^. The neutrino flux from clusters obtained in ref. ^13^ is comparable with observations by the IceCube Neutrino Observatory^2,17^. Most of the contribution to the total flux comes from clusters at redshift *z* ≤ 0.3 with masses *M* ≳ 10^14^*M*_⊙_.

## Results

We inject CRs with minimum energy of 100 GeV, such that we can study gamma-ray energies down to a few 10 GeV. The CRs can escape more easily from the regions with lower densities and magnetic-field strengths in the outskirts of the clusters, which decreases the gamma-ray flux. In Supplementary Fig. S4 of the Supplementary Material, we show the gamma-ray flux collected at the edge of individual clusters, produced by CR sources in different locations inside them. We find that the flux is one-order of magnitude larger when the source is located in the central region than in the edge of the cluster. For this reason, in order to compute the integrated contribution from all clusters in different redshifts below, we consider only the dominant contribution, i.e. from CR sources in the central region of the clusters.

**Fig. 1:**
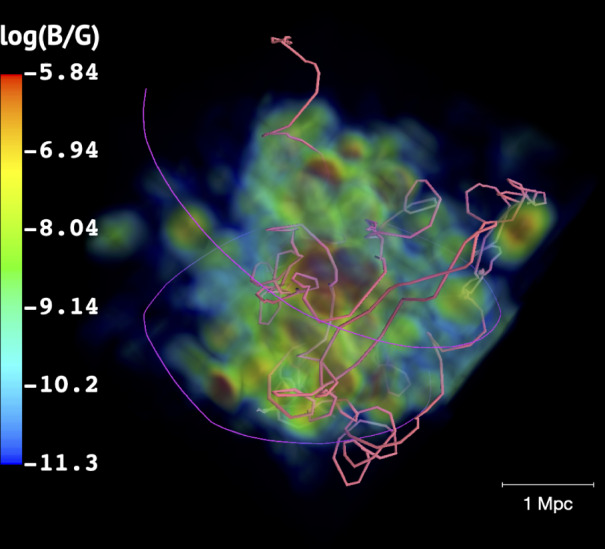
Trajectories of CRs through a cluster of mass ~10^15^ *M*_⊙_ selected from our background simulation. The map depicts the magnetic field intensity distribution in the cluster. The thick (pink) line corresponds to a CR with energy of 10 PeV, and the thin (purple) line to a CR with energy 500 PeV.

The mass range of clusters in our background simulation is 10^12^ ≲ *M*/*M*_⊙_ < 5 × 10^15^ and clusters with masses ≲10^13^ *M*_⊙_ barely contribute to the high-energy gamma-ray flux. This occurs due to the lower interaction rate between CRs and the intracluster environment, which is a consequence of the interplay between the Larmor radius, determined by the magnetic field, and the cluster size (see Supplementary Material for a detailed discussion). Also, massive clusters (≳10^15^ *M*_⊙_) exist mostly at low redshifts *z* ≲ 1, being rare at high redshifts. Therefore, the major contribution to the total flux comes from clusters in the mass range 10^13^ ≲ *M*/*M*_⊙_ ≲ 10^15^ (see Supplementary Fig. S5). Figure 1 illustrates the propagation of two CRs within a cluster of our background simulation.

**Fig. 2:**
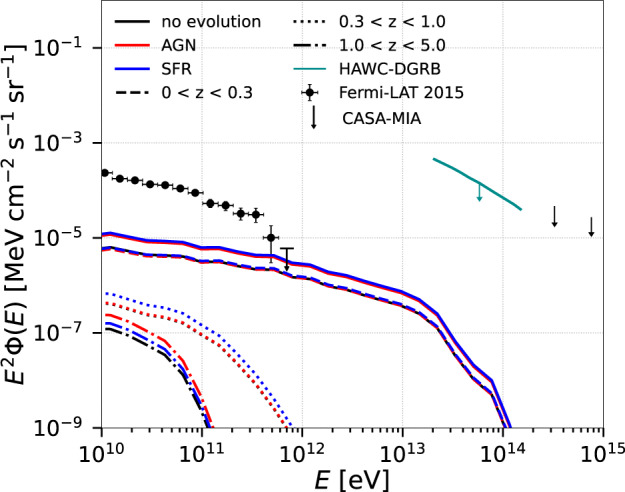
Gamma-ray flux from clusters at different redshift intervals. Total flux of gamma rays for *α* = 2.3 and ${E}_{\max }=1{0}^{17}$ eV over the entire redshift range (solid lines) and also for different redshift intervals (dash-dotted, dotted, and dashed). The solid lines are the sum of dashed, dotted and dashed-dotted lines. The figure also compares the flux including the separated contributions of the evolution of the CR sources (AGN and SFR) with the flux when there is no source evolution. For comparison, the observed flux by Fermi-LAT is depicted (error bars correspond to the total uncertainties, statistical and systematic)^66^, as well as the upper limits obtained by HAWC (95% confidence level)^31^ and CASA-MIA (90% confidence level)^32^ experiments.

**Fig. 3:**
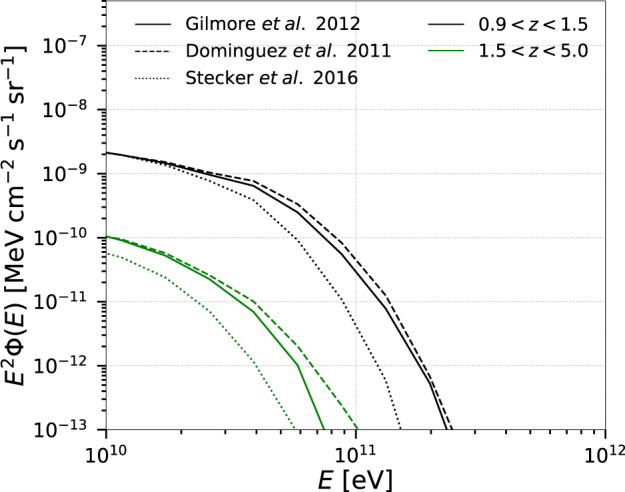
Effect of EBL attenuation on the gamma-ray flux for two redshift intervals and three different EBL models^20–22^. This figure indicates that the EBL flux attenuation is more prominent at high redshifts and sensitive to the adopted EBL model. The flux is plotted for *α* = 2.3 and ${E}_{\max }=1{0}^{17}$ eV.

**Fig. 4:**
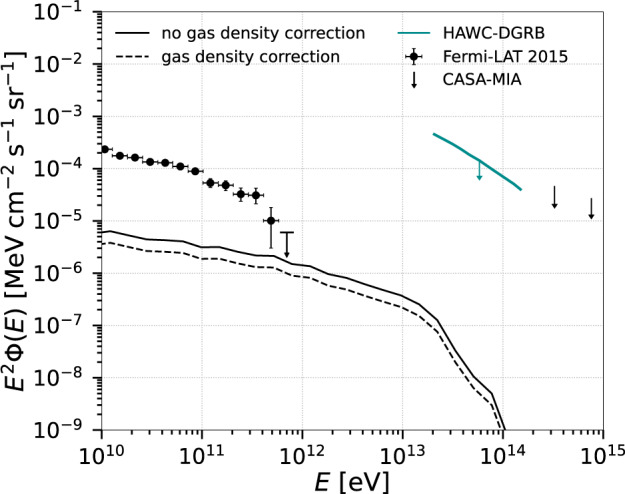
Total gamma-ray flux for *α* = 2.3 and ${E}_{\max }=1{0}^{17}$ eV over the entire redshift range as, in Fig. 2 (solid black line). It is compared with the total gamma-ray flux that we obtain when accounting for the gas loss of the clusters due to star formation and AGN feedback (black dashed line). The figure also shows the DGRB observations from Fermi LAT (error bars correspond to the total uncertainties, statistical and systematic)^66^, as well as the upper limits obtained by HAWC (95% confidence level)^31^ and CASA-MIA (90% confidence level)^32^ experiments.

**Fig. 5:**
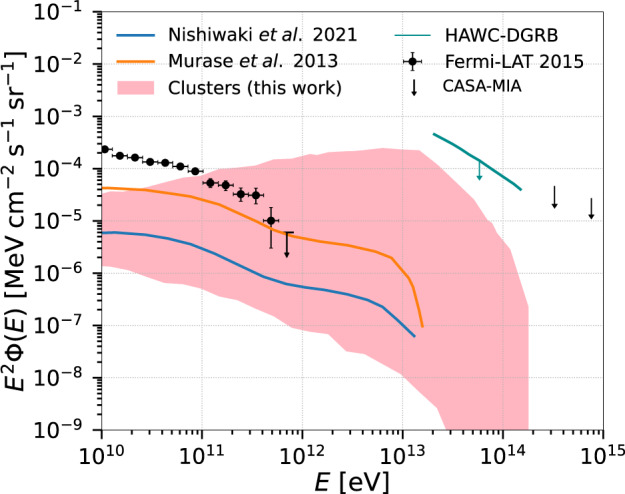
Integrated gamma-ray flux from the entire population of clusters. The pink shaded region represents the integrated gamma-ray flux obtained in this work for ${E}_{\max }=1{0}^{16}-1{0}^{17}$ eV and spectral index *α* = 1.5 − 2.5, as well as all source evolutions considered (AGN, SFR, and no evolution). This is compared with the total gamma-ray flux from clusters obtained in previous works^6,41^, and also with the DGRB observations from Fermi-LAT (error bars correspond to the total uncertainties, statistical and systematic)^66^, as well as the upper limits obtained by HAWC (95% confidence level)^31^ and CASA-MIA (90% confidence level)^32^ experiments.

In Figs. 2–5 we present the integrated gamma-ray spectrum from all clusters for *z*≤5.0, propagated up to the Earth. The total flux (Φ) was computed as follows:

1 $${E}_{{{{{{{{\rm{obs}}}}}}}}}^{2}\Phi ({E}_{{{{{{{{\rm{obs}}}}}}}}})=	\int\nolimits_{{z}_{\min }}^{{z}_{\max }}\,{{\mbox{d}}}z\int\nolimits_{{M}_{\min }}^{{M}_{\max }}{{\mbox{d}}}M\frac{{{\mbox{d}}}N}{{{\mbox{d}}}M}{E}^{2}\frac{{{\mbox{d}}}\,\dot{N}(E/(1+z),M,z)}\,{{{\mbox{d}}}\,E}\\ 	 g({E}_{{{{{{{{\rm{obs}}}}}}}}},E,z)\left(\frac{{\psi }_{{{{{{{{\rm{ev}}}}}}}}}(z)f(M)}{4\pi {d}_{L}^{2}(z)}\right)$$

where the number of clusters per mass interval *dN*/*dM* was calculated from our background simulation (see Supplementary Fig. S1), *g*(*E*_obs_, *E*, *z*) accounts for the interactions of gamma rays with energy *E* arriving with energy *E*_obs_ undergoing interactions during their propagation in the ICM and the intergalactic medium (IGM), *ψ*_ev_(*z*) is a function that describes the cosmological evolution of the emissivity of the CR sources (AGN, SFR, or none; see Eqs. (E1) and (E2) of the Supplementary Material), the quantity ${E}^{2}\,d\dot{N}/dE$ denotes the gamma-ray power computed from the simulation, *d*_*L*_ is the luminosity distance, and *f*(*M*) is a factor of order unit that corrects the flux by the amount of gas that is removed from the clusters due to stellar and AGN feedback. We note that the number of clusters per mass interval we obtained from our MHD cosmological simulation at different redshifts is comparable with results from other large-scale cosmological simulations^14–16^ and predictions from observations^18,19^ (see Supplementary Fig. S1).

The universe is believed to be isotropic and homogeneous at very large scales. Therefore, for the propagation of gamma rays from the clusters to Earth, we assumed a nearly uniform distribution of sources in comoving coordinates.

Figure 2 depicts the total flux for different redshift intervals: *z* ≤ 0.3, 0.3 < *z* ≤ 1.0, and 1.0 < *z* ≤ 5.0. A representative spectral index *α* = 2.3 and a maximum energy ${E}_{\max }=1{0}^{17}$ eV are used for this evaluation (see also Figs. 3 and 4). The dominant contribution to the total flux of gamma rays comes from sources at low redshifts (*z* ≲ 0.3), for which the effect of the EBL attenuation is less pronounced. This effect is more prominent at higher redshifts and also depends on the EBL model adopted^20–22^ (see Fig. 3, and Supplementary Fig. S7 of the Supplementary Material). Figure 2 shows the results for the EBL model from ref. ^20^, which predicts a slightly larger gamma-ray cut-off energy for the flux. Also, our treatment of the pp-interactions^23,24^ is only an approximation and contains uncertainties due to the unknown pp cross-section at energies beyond the reach of the LHC^25^.

Figure 2 also highlights the effects of the evolution of the CR sources on the gamma-ray flux, distinguishing the separated contributions of AGN and SFR, following the same procedure as in refs. ^13,26^. We find that an AGN-type evolution enhances the diffuse gamma-ray flux at high redshifts (*z* ≳ 1.5) compared to scenarios wherein the sources evolve as the SFR (or without any evolution). On the other hand, these contributions are both comparable at low redshifts (*z* ≲ 0.3) which in turn, provide the dominant contribution to the total gamma-ray flux.

We further notice that the flux of gamma rays above energies ~10^12^ eV can also be attenuated by interactions with the local optical and infrared photon fields of clusters, in addition to the EBL. Nevertheless, this effect is more dominant for sources at redshift *z* ≳ 0.3 as discussed in ref. ^27^. In our case, the major contribution corresponds to sources at *z* ≲ 0.3. Therefore, we expect that this interaction channel has likely a minor impact on our results.

As remarked, our MHD simulations do not include radiative-cooling, or the amount of gas that is converted into stars or removed from the clusters due to stellar and AGN feedback. This implies a slight overestimation of the density in the structures, especially for clusters of mass ≲10^14^ *M*_⊙_ (see refs. ^28,29^). Based on observational results^30^, we have also estimated the total gamma-ray flux taking into account the expected decrease of the gas density as a function of the cluster mass. In Fig. 4 we recalculated the total diffuse gamma-ray flux (black dashed line) considering correction factors *f*(*M*) ~ 0.95 for clusters with *M* ≳ 10^15^ *M*_⊙_, *f*(*M*) ~ 0.8 for *M* ≳ 10^14^ *M*_⊙_, *f*(*M*) ~ 0.3 for *M* ≳ 10^13^ *M*_⊙_, and *f*(*M*) ~ 0.3 for *M* ≳ 10^12^ *M*_⊙_, following ref. ^30^. A comparison between the dashed and solid black lines of Fig. 4 indicates a small reduction of the flux by at most a factor about 2.

The results for different combinations of the CR cutoff energy and spectral index are presented in Fig. 5. The shaded region shows the total flux of gamma rays for all clusters from the entire redshift range 0 < *z* ≤ 5.0, calculated for *α* = 1.5 − 2.5 and ${E}_{\max }=1{0}^{16}-1{0}^{17}\,\,{{\mbox{eV}}}$, including feedback by AGN and SF, and CR source evolution. The observed DGRB flux by Fermi-LAT, and the upper limits obtained by the currently operating HAWC^31^ and by the CASA-MIA experiment^32^, are also shown. For energies greater than ≳100 GeV, our simulations indicate that galaxy clusters can contribute substantially to the DGRB measured or constrained by these experiments. This contribution amounts for up to 100% of the observed flux by Fermi-LAT, for spectral indices *α* ≲ 2 and maximum energy ${E}_{\max }\gtrsim 1{0}^{17}$ eV. This also clearly explains the apparent flatness of the spectrum up to about 1 TeV (see also Supplementary Figs. S8 and S9 of the Supplementary Material).

### Discussion

The spectral indices considered here are consistent with the universal CR model^33^ used by Fermi-LAT to explore the CR induced gamma-ray emission from clusters^34^, and by H.E.S.S. for the Coma cluster (*α* = 2.1 − 2.4)^35^, while the ${E}_{\max }$ range is compatible with the fact that the clusters can confine mainly CRs with energies *E* ≲ 10^17^ eV^13,36^.

Note that the slope of the integrated gamma-ray flux is strongly influenced by the spectral parameters of the injected CRs. Therefore, when considering potential values for the CR spectral index, it is also important to discuss the corresponding particle acceleration mechanism(s). If CRs are accelerated by the same processes that produce ultra-high-energy CRs (UHECRs), phenomenological fits^26,37,38^ to the UHECR data favor very hard spectra, with possible spectral indices extending as low as *α* < 0 in some cases (for *E*^−*α*^). Such scenarios might seem surprising, at first, but there are sound explanations that include magnetic field effects, plasma instabilities, re-acceleration, magnetic reconnection, interactions in the sources, etc (see, e.g., refs. ^3,4,39,40^ for an overview on some of these mechanisms). Naturally, the spectral properties of CRs injected in the ICM in the PeV-EeV range do not need to be the same as the UHECRs, but it is reasonable to expect a connection. Therefore, even hard spectral indices are theoretically possible. Note that if the CRs responsible for producing the gamma rays are accelerated not by CR sources embedded in clusters but via accretion or merger shocks, for example, then softer spectra (*α* ~ 2.0 − 2.3) are expected. In Supplementary Fig. S8 of the Supplement Material, we show the gamma-ray flux for different combinations of the parameters $\alpha \,\,{{\mbox{and}}}\,\,{E}_{\max }$.

In Fig. 5, the gamma-ray flux we obtained from the entire population of clusters is also compared with the expected one from Coma-like clusters^6^, and that obtained in ref. ^41^. In ref. ^41^, they estimated the gamma-ray flux from clusters using a purely hadronuclear scenario (*pp*-interaction) claiming that these sources would contribute to the DGRB with at least 30–40%, or even 100% if the spectrum is soft (*α* ≳ 2.2). In comparison with the estimated spectrum for Coma-like clusters^6^, our gamma-ray flux is a little higher. In both studies^6,41^, besides the oversimplified ICM magnetic-field and density distributions, assumed to have radial profiles, they did not account for the contributions from clusters of mass ≲10^14^ *M*_⊙_. In Coma-like clusters^6^, where masses are of the order of 10^15^ *M*_⊙_, the average density is ~10^−6^ Mpc^−3^, but it is ~10^−4^ Mpc^−3^ for cluster masses of a few 10^14^ *M*_⊙_ (as considered in refs. ^6,41^), and can be even larger for masses <10^14^ *M*_⊙_, as predicted by large scale cosmological simulations^14–16^ and obtained in our own MHD simulations. Because we are considering here the entire mass range (10^12^ ≤ *M*/*M*_⊙_ < 5 × 10^15^), the density is higher by an order of magnitude, and this is the main difference between ours and these previous studies^6,41^.

Another study^42^ estimated the flux using a simple relation between the gamma-ray luminosity and the cluster mass. They constrained the radio-loud cluster count from observations by the Radio Astronomy Observatory Very Large Array sky survey^18,43^ and also assumed that the radio luminosity scales linearly with the hadronic high-energy emission. Their results are also comparable with ours.

Though individual source populations such as blazars^44,45^, misaligned-AGNs^46^ and star-forming galaxies (SFGs)^47^ can contribute to a fairly large fraction to the DGRB for energies below TeV^48,49^ (see Supplementary Fig. S9 of the Supplementary Material), our results demonstrate that the cumulative gamma-ray flux from clusters can dominate over the integrated contribution of individual classes of unresolved sources, at energies ≳100 GeV. The implications of our calculations are extremely important considering that the contribution from clusters is guaranteed if high-energy CRs are present in the ICM.

As shown in Fig. 5, our results are compatible with upper limits evaluated by HAWC^31^. A similar estimate has yet to be performed by other facilities like the LHAASO^50^ or the forthcoming CTA^51^. Nevertheless, considering the sensitivity curves for point sources obtained in both cases^50,51^, the gamma-ray flux we derived has likely the potential to be detected by these facilities too (see also Supplementary Fig. S9 and the discussion therein).

Future more realistic MHD cosmological simulations that account directly for the CR sources distribution, evolution, and feedback^52,53^ may allow to constrain better the contribution of clusters to the DGRB. Furthermore, the effects of unknown magnetic fields of the diffuse IGM on the gamma-ray cascading may also change our results (see discussion in the Supplementary Material).

**Fig. 6:**
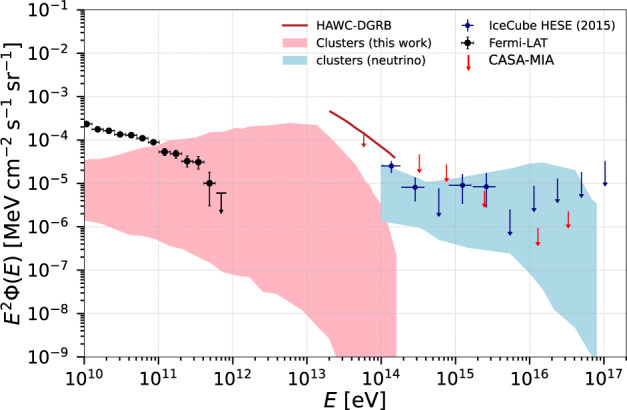
Multi-messenger emission from clusters of galaxies. High-energy neutrinos (blue band) (obtained by^13^, error bars in IceCube data correspond to the 68% confidence intervals^67^) and gamma rays (pink band) from the entire population of galaxy clusters obtained in this work. The gamma-ray flux is compared with the DGRB observed by Fermi-LAT (error bars correspond to the total uncertainties, statistical and systematic)^66^, and the upper limits by HAWC (95% confidence level)^31^ and CASA-MIA (90% confidence level)^32^.

Figure 6 summarizes our findings, together with those from ref. ^13^. It shows both the high-energy gamma-ray and neutrino emission from the entire population of clusters up to redshift *z* ≤ 5.0, assuming CR sources embedded in clusters. As we see, the neutrino flux we obtain is comparable with the diffuse neutrino background observed by IceCube for CR spectral index *α* = 1.5 − 2.5 and maximum energy 10^16^ − 10^17^ eV. A recent analysis by the IceCube Collaboration^17^ found that less than ~77% of the total diffuse neutrino flux could be due to clusters. While this could, at first glance, seem in conflict with our results, we note that changes in the parameters of our analysis such as the total CR luminosity or the distribution of CR sources within the cluster could reduce our estimate. The same is true for the DGRB predictions. Therefore, the link established by Fig. 6 between the diffuse gamma-ray and the diffuse neutrino backgrounds, should be interpreted minding these caveats.

Our results were obtained through the most detailed simulations to date of three-dimensional particle transport in cosmological environments. Combined with the other known components of the DGRB, our results strongly constrain the fraction of the diffuse flux that could be ascribed to unknown components such as the elusive dark matter. Moreover, it establishes a clear connection between the fluxes of two messengers, neutrinos and gamma rays, which, combined, enables us to indirectly study CRs in clusters even if they are not directly observable.

## Methods

We describe the ICM through 3D-MHD smoothed-particle-hydrodynamical (SPH) cosmological simulations employing the GADGET code^54,55^, within a sphere of radius 110 Mpc around the Milky Way^7^. The simulations extend up to a redshift of *z* ≃ 5 and contain clusters with masses 10^12^ < *M*/*M*_⊙_ < 10^15.5^. We consider here seven snapshots at redshifts 0, 0.05, 0.2, 0.5, 0.9, 1.5, and 5.0. For clusters in this mass range, the corresponding luminosity interval is about (10^42^ − 10^46^) erg s^−1^^56^. These values are used to compute the gamma-ray fluxes shown throughout this work. The magnetic-field strength varies between 10^−11^ G and 10^−5^ G approximately, which is in reasonable agreement with the expected field strengths from observations of different clusters of galaxies^57^. Feedback from active galactic nuclei (AGN) and star formation (SF) are not directly included in these MHD cosmological simulations, but the evolution effects of these potential CR sources on the flux of gamma rays is accounted for with a redshift-dependent profile, as in refs. ^13,26,58^ (see Eqs. (E1) and (E2) of the Supplementary Material). A flat ΛCDM universe model is assumed, with the corresponding cosmological parameters given by *h* ≡ *H*_0_/(100 km s^−1^ Mpc^−1^) = 0.7, Ω_*m*_ = 0.3, Ω_Λ_ = 0.7, and the baryonic fraction Ω_*b*_/Ω_*m*_ = 14%. The maximum resolution in our SPH simulations is ~10 kpc (see refs. ^7,13^ and also page 1 of the Supplementary Material for details).

We are interested in high-energy gamma rays with *E* ≳ 10 GeV whose origin is more uncertain (see Supplementary Fig. S9 of the Supplementary Material) and thus consider CRs with energies 10^11^ ≤ *E*/eV ≤ 10^19^. The energy around 10^19^ eV can be achieved by primary sources inside a cluster, such as AGNs^2,59^. For magnetic fields of *B* ~ 1 μG, the Larmor radius of CRs with *E* ~ 10^19^ eV is *r*_L_ ~ 10 kpc, so that they cannot remain trapped within clusters for too long. On the other hand, CRs with lower energies remain confined, producing secondaries due to interactions with the ICM gas and the bremsstrahlung radiation, as well as with the CMB and the EBL^6,13,36,59,60^.

We explore the propagation of CRs in the simulated background of clusters using the CRPropa code^61,62^. The propagation has two steps and we assume that the CRs are predominantly composed by protons, since we expect much smaller contribution from heavier elements^12^ (see page 4 of Supplementary Material). In the first step, we compute the gamma-ray flux produced by CR interactions in the clusters by considering all relevant interactions that generate both electrons and photons, namely: photopion production, Bethe-Heitler pair production, pair production, inverse Compton scattering, and proton-proton (pp) interactions. In addition, we take into account the energy losses due to the adiabatic expansion of the universe and due to synchrotron emission, although these only contribute to the electromagnetic flux at energies much lower than our energy of interest (*E* ≳ 10 GeV). For more details on how CRs were propagated, see the Supplementary Materials (Supplementary Figs. S2 and S3). We find that the interactions of the CRs with the cluster gas and the CMB are the dominant channels for producing the secondaries^13^. In the second step, we perform the propagation of the gamma rays collected at the boundary of the clusters to Earth. We consider the electromagnetic cascade process initiated by these gamma rays both in the ICM and in the intergalactic medium, including inverse Compton scattering, single, double, and triple pair production, with the CMB, the EBL^20^, and the radio background^63^ (see Supplementary Fig. S3 in Supplementary Material). We did not consider the effects of intergalactic magnetic fields outside the cluster in this step, since they are highly uncertain^64^ and are not expected to majorly affect the gamma-ray flux at energies above 100 GeV^65^.

To compute the gamma-ray flux we have followed the same procedure given in ref. ^13^ and considered that 1% of the cluster luminosity goes into CRs, which is consistent with Fermi-LAT predictions^34^. We only considered the contribution of CRs with energies above 100 GeV approximately, although we did consider the whole energy range, starting from 1 GeV, to normalize the total energy of the simulation to the cluster luminosity, as explained on page 4 in the Supplementary Material. Also, in Supplementary Fig. S6 of the Supplementary Material, we compare the gamma-ray flux for different values of this luminosity fraction and the results indicate a variation much less than an order of magnitude.

## Supplementary information

Supplementary Information

## Data Availability

The datasets generated during and/or analyzed during the current study are available from the corresponding author upon request. Source data are provided with this paper.

## Source data

Source Data

## Code availability

The numerical codes used to generate results that are reported in the current study are available upon request.
