# Supplementary material for: The diffuse gamma-ray flux from clusters of galaxies
Source: Nat Commun. 2023 Apr 29;14:2486. doi: 10.1038/s41467-023-38226-w (PMC10148864; doi:10.1038/s41467-023-38226-w)
Supplement: Supplementary file 1 — Supplementary Information [file 41467_2023_38226_MOESM1_ESM.pdf]

The Diffuse Gamma-Ray Flux from Clusters of Galaxies

Saqib Hussain[1,2*], **Rafael Alves Batista**[3,4], **Elisabete M. de Gouveia Dal Pino**[1], **and Klaus Dolag**[5,6]

[1]Institute of Astronomy, Geophysics and Atmospheric Sciences (IAG), University of São Paulo (USP), R. do Matão, 1226, 05508-090, São Paulo, Brazil
[2]Gran Sasso Science Institute, Via Michele Iacobucci, 2, 67100 L'Aquila, Italy, Email: saqib.hussain@gssi.it
[3]Instituto de Física Teórica UAM-CSIC, C/ Nicolás Cabrera 13-15, 28049 Madrid, Spain
[4]Departamento de Física Teórica, Universidad Autónoma de Madrid, M-15, 28049 Madrid, Spain
[5]University Observatory Munich, Scheinerstr. 1, 81679 München, Germany
[6]Max Planck Institute for Astrophysics, Karl-Schwarzschild-Str 1, 85741 Garching, Germany

Supplementary Material

Cosmological simulations. To calculate the contribution of galaxy clusters to the diffuse gamma-ray background (DGRB), we employed three-dimensional cosmological MHD simulations[1] obtained with the GADGET code[2,3]. They cover a large volume (a sphere of radius ~ 110 Mpc) and a redshift range $z \simeq 0-5$, and contain several clusters with masses $10^{12} < M/M_{\odot} < 10^{15.5}$. At $z = 0$, these simulations reproduce quite well the distribution of nearby clusters, including Virgo, Perseus, and Coma, which are within 100 Mpc away from Earth approximately. The number of clusters per mass interval we obtained at different redshifts is comparable with results from other large-scale cosmological simulations[4–6] (see Fig. S1), and with predictions from observations[7,8].

Cosmic-ray propagation in clusters. Using the sample of individual clusters obtained from the cosmological simulations, we considered sources of high-energy cosmic rays (CRs) embedded in these structures to compute the associated gamma-ray fluxes. As explained in the Methods of the main text, we used the CRPropa code[9,10] for these calculations considering all relevant CR interactions that generate both electrons and photons, namely: photopion production, Bethe-Heitler pair production, and proton-proton (pp) interactions. The latter is not natively implemented in the code, so we employed an external CRPropa module described in ref.[11], which uses the cross section for pp interactions as parameterised in ref.[12], given by:

$$\sigma_{\rm pp}(E) = \left[30.7 - 0.96\log\left(\frac{E}{E_{\rm th}}\right) + 0.18\log^2\left(\frac{E}{E_{\rm th}}\right)\right]\left[1-\left(\frac{E_{\rm th}}{E}\right)^{1.9}\right]^3 \text{ mb} \qquad \text{(E1)}$$

with E denoting the kinetic energy for a threshold energy $E_{\rm th} \equiv 2m_\pi c^2 + m_\pi^2 c^2/2m_p$. Here m_π denotes the mass of the corresponding pion, and m_p the mass of a single proton. In addition, we have taken into account the energy losses due to the adiabatic expansion of the universe and synchrotron emission. We did not make any approximations to describe the properties of the ICM. Instead, we used the background density, temperature, and magnetic fields, directly from the MHD simulations. The temperature distribution in the clusters allowed us to derive the bremsstrahlung radiation field, which we used to extend CRPropa's pre-computed tables of interaction rates to include interactions with the cluster environment. We also used the distributions provided in CRPropa for the other background photon fields, namely the extragalactic background light (EBL)[13–15] and the cosmic microwave background (CMB). The mean free paths (MFPs)

for a CR undergoing the aforementioned processes are shown in Fig. S2, left panel. For reference, we also show the expected trajectory length of CRs propagating in individual clusters with different masses (Fig. S2, right panel).

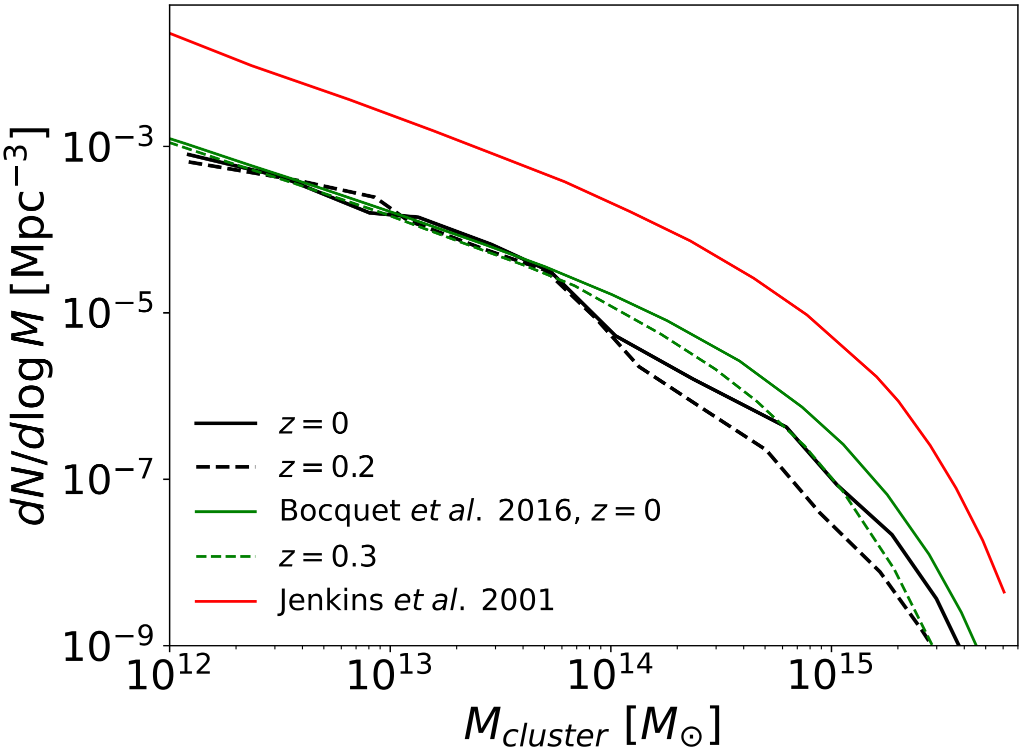

Figure S1. Cluster density: Black lines represent the number of clusters per mass interval in our cosmological simulation for different redshifts. For comparison, results from other large-scale cosmological simulations are also shown as green[6]and red lines[4]. We note that in ref.[4] (red line) it is presented the total count of clusters as a function of mass starting at redshift $z = 14$ up to $z = 0$. This explains the difference with regard to the other curves.

Magnetic confinement of CRs. The transport of CRs inside clusters is highly dependent on their masses. The more massive clusters $\gtrsim 10^{14}\ M_\odot$ can confine CRs of higher energy for a time longer than the less massive (and smaller) ones. This is consistent with the increase of the particle's Larmor radius with energy while moving inside the cluster. As the cluster mass increases, the transport of CRs changes from diffusive to semi-diffusive or ballistic. This regime change depends on the diffusion coefficient which, in the simplest case, is $D = \langle r^2 \rangle / 6t$, wherein r is the displacement and t the time. Considering a typical size of 1 Mpc and assuming the cluster to exist roughly for a time comparable to the age of the universe, it is possible to obtain an order-of-magnitude estimate of the diffusion coefficient associated to CR confinement/escape, which is $D \sim 10^{27}\ \mathrm{m^2\,s^{-1}}$. Comparing this with the Larmor radius of CRs with energy $E \sim 10^{17}$ eV, we conclude that in the central regions of the cluster ($r \lesssim 500$ kpc) propagation is diffusion-dominated, whereas in the outskirts CRs can escape the environment. Moreover, high-energy CRs ($E \gtrsim 10^{18}$ eV) propagate (quasi-)balistically. These results agree with those obtained from a simple estimate of the confinement time of a CR, which can be obtained from the trajectory length (ℓ): $t \simeq \ell / c \simeq 10^3\ \mathrm{Mpc}/c \sim$ Gyr, wherein c denotes the speed of light. Note that this is comparable with

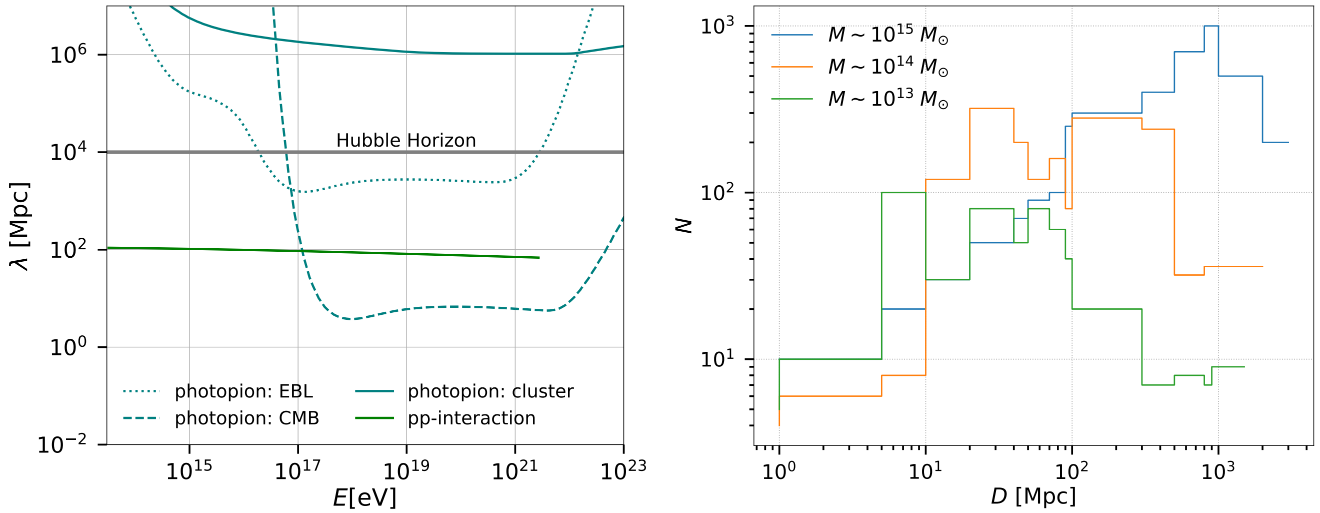

Figure S2. MFP and trajectory lengths of protons. Left panel: MFP for the processes affecting CRs, namely photopion production, and proton-proton interactions. The target photon fields are the CMB, the EBL (from ref.[14]), and the volume-averaged bremsstrahlung radiation for a cluster of mass $M = 10^{15} M_\odot$. Right panel: trajectory lengths of CRs emitted at the centre of clusters of mass $10^{13}\mathrm{M}_\odot$, $10^{14}\mathrm{M}_\odot$, and $10^{15}\mathrm{M}_\odot$ with a spectrum E^{-1}, for $10^{14} \leq E/\mathrm{eV} \leq 10^{16}$.

the diffusive escape time found in other works[16,17]. For instance, the acceleration time of a CR up to about 10^{18} eV in a magnetic field of the order of 10^{-6} G for a shock in a cluster is of the order of Gyr[18], which is comparable with the diffusive escape time from the acceleration region[16].

Gamma-ray and electron propagation in clusters. Electrons and photons produced through the processes described in the previous paragraph also undergo interactions, namely: pair production, inverse Compton scattering, double pair production, and triplet pair production. These interactions were taken into account assuming the omnipresent cosmological backgrounds (CMB and EBL), in addition to the ICM photon field due to the bremsstrahlung. The MFPs for these processes are shown in Fig. S3 for both electrons (left panel) and photons (right panel). Note that high-energy photons can, in principle, interact with the gas pervading the ICM, which could lead to "inverse photopion production". Nevertheless, this channel of interaction is small, as shown on the right panel of Fig. S3, so it was thus neglected.

The effects of CR source evolution. The evolutionary history of galaxy clusters implies a constantly changing ICM which, in turn, affects the propagation of CRs and its subsequent gamma-ray emission. For this reason, we have considered a few different scenarios for the evolution of the CR sources embedded in the cluster. This is parametrised through a redshift-dependent function $\psi(z)$. For CR sources following the star-formation rate (SFR), we employ the following expression[19,20]:

$$\psi_{\mathrm{SFR}}(z) = \frac{1}{B} \begin{cases} (1+z)^{3.4} & \text{if } z \leq 1 \\ (1+z)^{-0.3} & \text{if } 1 < z \leq 4 \\ (1+z)^{-3.5} & \text{if } z > 4\,. \end{cases} \tag{E2}$$

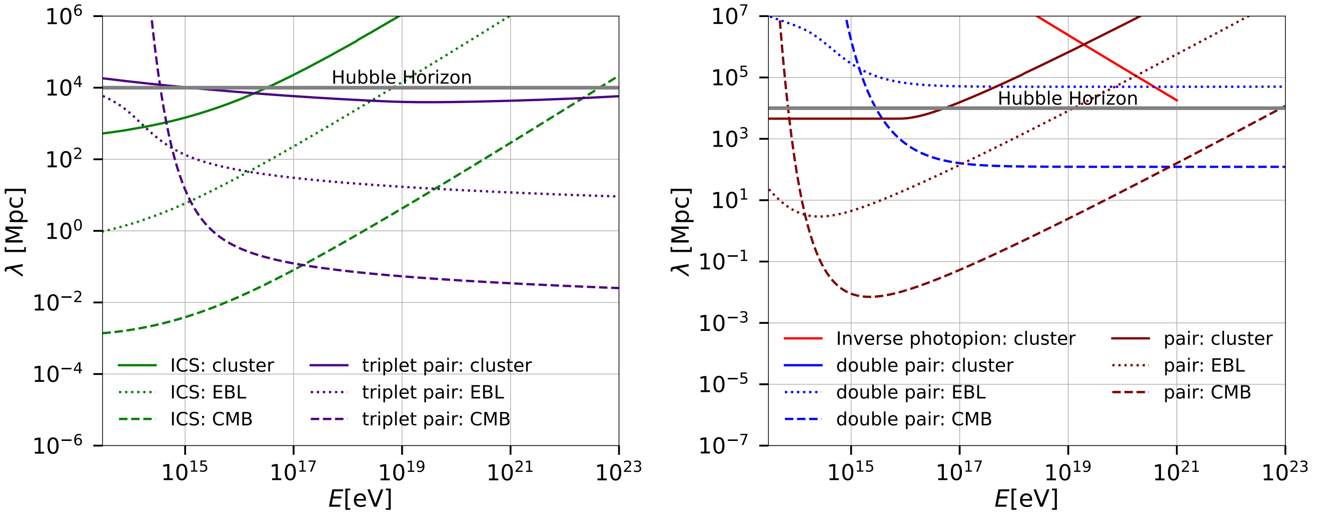

Figure S3. MFPs of electrons and photons: Shown are the processes affecting high-energy electrons (inverse Compton scattering (ICS) and triplet pair production), and photons (pair production and double pair production). The target photon fields are the CMB, the EBL (from ref.[14]), and the volume-averaged bremsstrahlung radiation for a cluster of mass $M = 10^{15} M_\odot$.

Assuming the CR source emissivity is driven by AGNs, the parametrization reads[19,20]:

$$\psi_{\mathrm{AGN}}(z) = \frac{1}{A} \begin{cases} (1+z)^{5.0} & \text{if } z \leq 0.97 \\ 10^{1.09}(1+z)^{1.33} & \text{if } 0.97 < z \leq 4.48 \\ 10^{6.66}(1+z)^{-6.2} & \text{if } z > 4.48\,. \end{cases} \tag{E3}$$

Here A and B are normalization constants in Equations (E3) and (E2), respectively.

The effect of star formation and AGN feedback. Our simulations are non-radiative and do not include the feedback by active galactic nuclei (AGNs) or star formation, which could reduce the gas density of the clusters and consequently, the gamma-ray flux. To investigate the relevance of these effects to our calculations we followed ref.[21], which evaluates how a density reduction factor $f(M,z)$ (Equation 1 in the main text) can be empirically constrained by observations of different clusters. We find that both effects above produce only minor modifications in the total flux, as shown in Figs. 2 and 5 of the main text.

The CR injection spectrum. The injection spectrum of CRs ($Q(E)$) is defined as:

$$Q(E) = \frac{dN}{dE} = Q_0 E^{-\alpha} \exp\left(-\frac{E}{ZR_{\mathrm{max}}}\right), \tag{E4}$$

wherein α is the spectral index, R_{max} is the maximum rigidity attainable by the CRs, and Z is the atomic number of the CR nucleus ($Z = 1$ here). To obtain the normalization constant, Q_0, we impose that the total CR energy must be a given fraction (f_{CR}) of the cluster energy, i.e.:

$$\int_{E_{\mathrm{min}}}^{E_{\mathrm{max}}} \mathrm{d}E\, E Q(E) = f_{\mathrm{CR}} E_{\mathrm{tot}}\,, \tag{E5}$$

where E_{tot} refers to the total energy of the cluster corresponding to a luminosity L_{tot}. Here the minimum and maximum energies, $E_{min} \simeq 1$ GeV and $E_{max} \simeq 10$ EeV, are essentially the rest mass of the CR and the maximum energy a CR could reach according to the model, respectively. The later, in particular, was conservatively chosen to be 10 EeV because already at much lower energies (about 0.1 EeV) CRs can escape clusters without effectively interacting with the ICM.

A note on the composition of the CRs. We assumed only proton composition of CRs, because we expect a much smaller contribution from heavier elements (see, e.g.,[22]), especially if they are produced in large-scale shocks. If there are heavier nuclei in clusters, they should be subdominant with respect to protons because CR acceleration depends on rigidity (energy over charge). Nevertheless, CR sources within clusters such as starburst galaxies have high supernova rates, and compact objects such as magnetars wherein heavier energetic CRs can be accelerated, such that the gamma-ray flux may change. It is also worth mentioning that gamma rays (and neutrinos) in general tend to be produced more through processes involving protons than nuclei. This is because hadronic gamma rays are created mostly through the decay of pions, and in the case of heavier CR nuclei, photodisintegration tends to dominate over pion-producing mechanisms. Furthermore, photons produced, for instance, by electron/positrons generated via nuclear beta decays (in the photodisintegration chain, for example) are generally not sufficient to lead to appreciable fluxes of high-energy photons.

The injected energy range of CRs is $10^{11} \leq E/\text{eV} \leq 10^{19}$, which leads to a peak of the integrated gamma-ray flux at energies around 10 GeV as shown in Figs. 2 and 4 of the main text (see also Fig. S7).

The gamma-ray flux from individual clusters. Our analysis involve computing first the gamma-ray flux emitted by individual clusters. This is shown in Fig. S4 for masses $M \sim 10^{15}\, M_\odot$ and $10^{14}\, M_\odot$. The figure also shows the dependence of the photon flux on the position of the CR sources inside the clusters. As expected, the photon production rate is smaller when the source is located farther away from the centre. Note that the central regions of clusters are more densely populated than the outskirts. Therefore, it is a reasonable approximation to consider all CR sources inside clusters to be at their centres, since the contribution of marginal sources is lower by nearly a ten-fold, as shown in Fig. S4. This implies that even under these assumptions the total gamma-ray flux would be overestimated by less than an order of magnitude.

A sanity check for our calculations is to compare the results for a few individual clusters selected from our simulations with observations. Fermi-LAT[23], for instance, obtained upper limits for the emission from three Abell clusters, A400, A1367, and A3112. At $E \sim 10$ GeV, the most stringent amongst these limits is $\simeq 4.4 \times 10^{-7}$ MeV cm^{-2} s^{-1}. Although Fig. S4 is for the flux at the edge of the cluster, without intergalactic propagation, its total gamma-ray energy is $E_{tot}^{sim} \lesssim 2 \times 10^{43}$ MeV, which is much less than the total energy inferred from observations, $E_{tot}^{obs} \sim 4 \times 10^{45}$ MeV, considering the cluster A400, distant approximately 100 Mpc from Earth. There is also a geometrical correction factor due to the fact that the simulated flux is divided by a solid angle, but this should not exceed one order of magnitude in the most conservative case. Therefore, this simple ballpark estimate confirms that our gamma-ray estimates for individual clusters are safely compatible with present-day observational constraints[23–25].

The integrated gamma-ray flux from different cluster mass ranges. In Fig. S5, we present the dependence of the gamma-ray flux on the mass of the clusters. The major contribution comes from clusters in the mass range $10^{13} \lesssim M/M_\odot \lesssim 10^{15}$.

The CR luminosity. Throughout this work, in order to compute the gamma-ray flux, we have considered that $f_{CR} \sim 1\%$ of the luminosity of a cluster goes into CRs, which is consistent with estimates from observations (see e.g. refs.[23,29]), as stressed in the main text. However, to illustrate the relevance of this

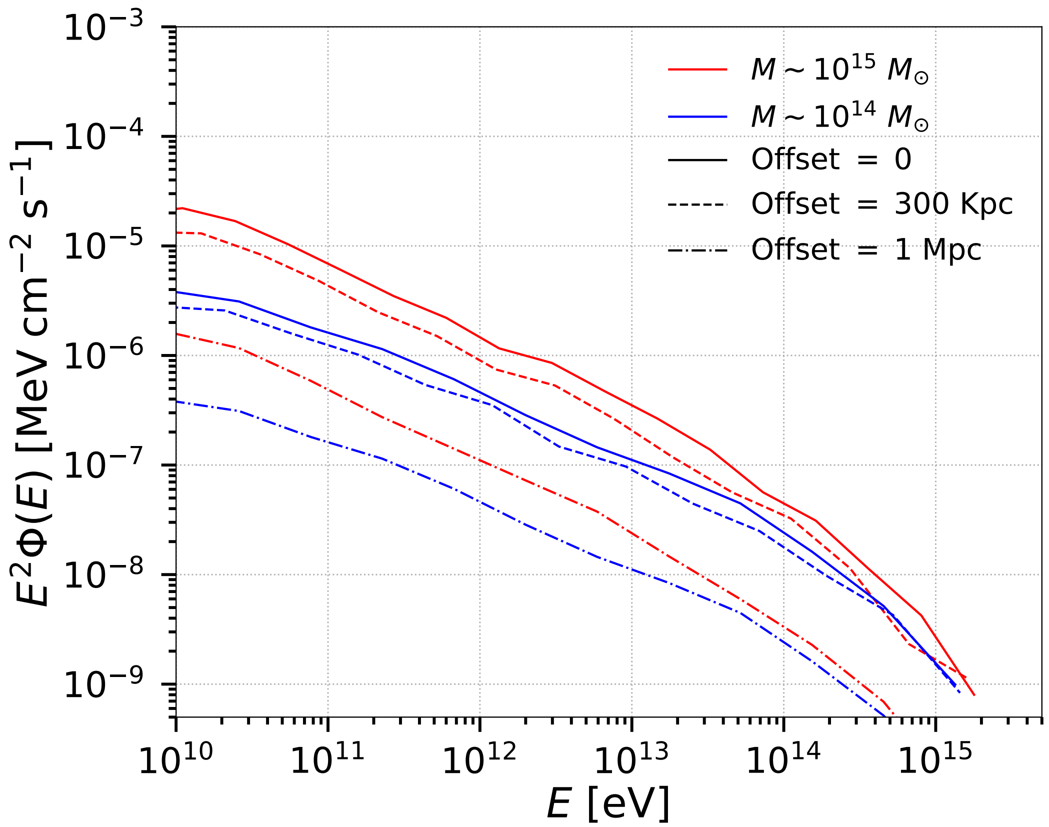

Figure S4. **Gamma-ray flux at the edge of individual clusters** (radius ~ 2 Mpc) of mass $M \sim 10^{15}\ M_\odot$ (red); and $10^{14}\ M_\odot$ (green), at redshift $z \sim 0$. We considered CR sources located at the center of the cluster (solid lines), at 300 kpc (dashed lines), and at 1 Mpc away from the center (dash-dotted lines). The spectral index of the CR spectrum has a power-law index $\alpha = 2.3$ and an exponential energy cut-off $E_{\mathrm{max}} = 10^{17}$ eV.

parameter, in Fig. S6, we show the gamma-ray flux spanning a range of values of this fraction, namely, $f_{\mathrm{CR}} \sim (0.5-5)\%$ of the luminosity of the clusters going into CRs. The results indicate that the variation is not substantial, i.e., it is at most of one order of magnitude, which is compatible with the linear dependence between flux and luminosity.

Integrated gamma-ray flux for different redshift intervals. As indicated in Fig. 2 of the main text, the major contribution to the integrated flux comes from CR sources at low redshifts $z \lesssim 0.3$, whose flux is less attenuated by interactions with the EBL. This suggests that the resulting spectral hardening is due to this low redshift contribution mostly. This result is reassured by Fig. S7, which shows the gamma-ray flux for all redshift intervals, and is complementary to Fig. 2 of the main text.

Integrated gamma-ray flux for different CR spectral parameters. In Fig. S8 we show the gamma-ray flux for different combinations of the parameters α and E_{max} of the CRs. The choice of this parametric range is discussed in detail in the main text.

Integrated gamma-ray flux for entire parametric space. As stressed previously, the CRs are injected with a minimum energy of 100 GeV, and our analysis of the gamma-ray flux produced by them in the clusters extends down to 10 GeV approximately. In any case, our main interest is the contribution of the

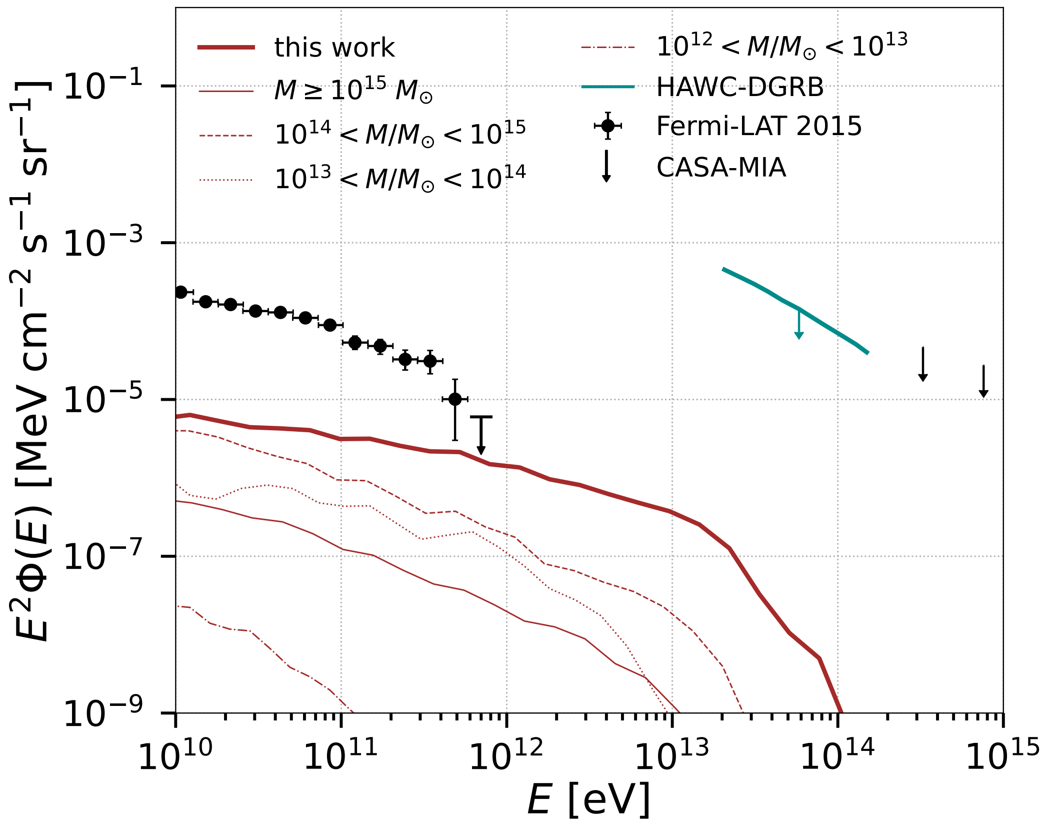

Figure S5. Contribution to the integrated gamma-ray flux from different cluster mass ranges. The flux is plotted for CR spectral parameters $\alpha = 2.3$ and $E_{\max} = 10^{17}$ eV. Fermi-LAT data for the DGRB (error bars correspond to the total uncertainties, statistical and systematic)[26], upper limits from HAWC (95% confidence level)[27] and CASA-MIA (90% confidence level)[28] are also shown for comparison.

clusters to the higher energies of the DGRB, whose origin is more uncertain, less constrained and, in principle, not explained by point sources or individual source populations, as shown in Fig. S9. We have also plotted in this figure point-like sensitivity curves of different gamma-ray observatories, which were rescaled by an appropriate angular factor. This is meant to be a reference only. There are experimental difficulties in measuring an all-sky flux with Cherenkov telescopes with relatively small fields of view such as CTA. The message intended is: if CTA could scan the whole sky and measure a diffuse flux of gamma rays, then the ideal curve obtained by a direct scaling of the point-source sensitivity would be the one shown. In fact, this figure evidences that the major contribution to the DGRB below about 400 GeV most probably comes from individual sources[30] such as blazars[31], AGNs[32], and SFGs[33]. But for energies greater than $\gtrsim$ 100 GeV, our simulations indicate that galaxy clusters can also contribute substantially to the DGRB. This contribution could amount to up to 100% observed flux by Fermi-LAT, for spectral indices $\alpha \leq 2$ and maximum energies $E_{\max} \geq 10^{17}$ eV.

Remarks on the interpretation of the results. Our goal in this work was not to fit the data observed by Fermi-LAT. Instead, we calculated the high-energy gamma-ray flux that can be produced by the entire galaxy cluster population, considering a reasonable set of free parameters, and performing the most detailed treatment so far employing 3D simulations (in contrast, many studies until now adopted a semi-analytic approach and/or simplified 1D calculations). Uncertainties in such determinations are always expected.

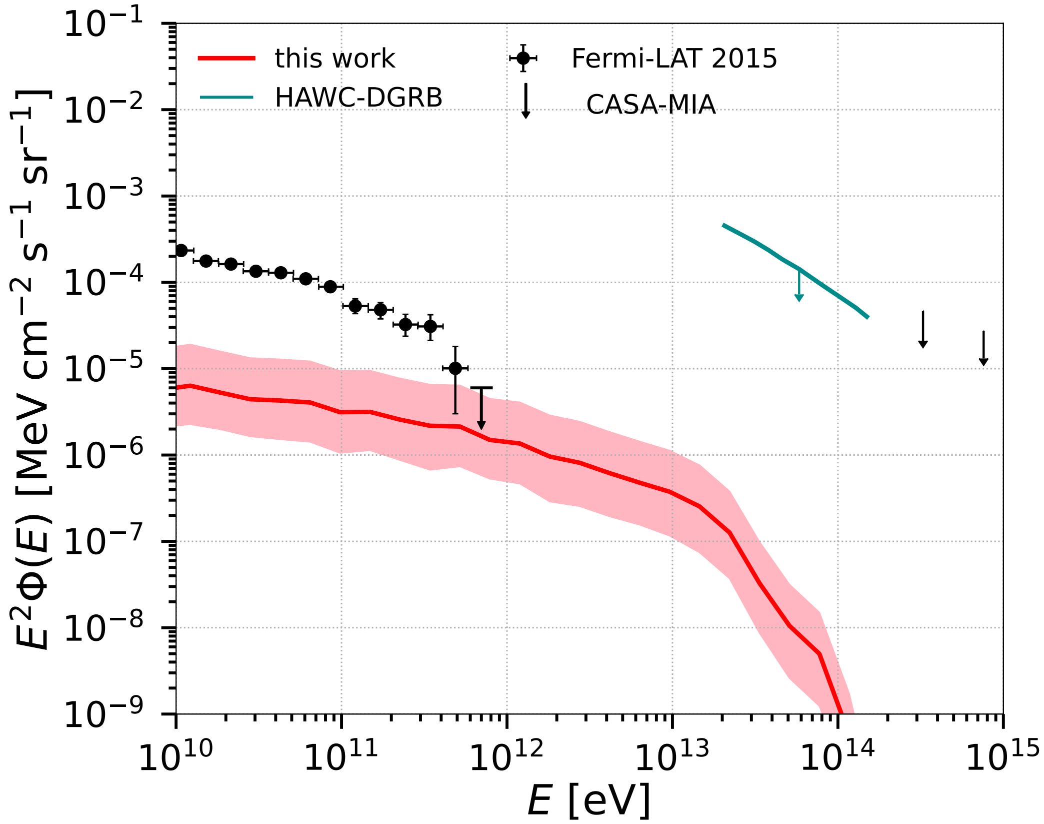

Figure S6. Integrated gamma-ray flux from clusters for different values of the fraction of the cluster luminosity that goes into CRs. The pink band corresponds to $(0.5-5)\%$ of this fraction and the red line corresponds to 1%. Here we assume a CR spectral index $\alpha = 2.3$ and a cutoff energy $E_{\rm max} = 10^{17}$ eV. Fermi-LAT data for the DGRB (error bars correspond to the total uncertainties, statistical and systematic)[26], upper limits from HAWC (95% confidence level)[27] and CASA-MIA (90% confidence level)[28] are also shown.

The question is whether they lead to order-of-magnitude changes in the results. To answer this question, we explored the parametric space of the potentially most influential quantities, i.e., we considered a fiducial range of the CR spectral parameters, which are all compatible with theoretical/observational expectations. Moreover, we employed a detailed treatment of intergalactic gamma-ray propagation, including EBL uncertainties (see Fig. 3 of the main text). Fermi observations and upper limits obtained from HAWC and CASA-MIA for the DGRB, depicted in Fig. 5 of the main text, and Fig. S8, clearly put constraints on the parametric space we swept. Our results turn out to be compatible with these constraints for spectral indices $\gtrsim 2.3$, considering our fiducial parameters. Therefore, though uncertainties remain, such as the determination of the effects of the still-unknown magnetic fields of the diffuse IGM on the gamma-ray cascading after emerging from individual clusters (as described in the main text), we believe we have covered most of the fundamental parametric space, thus constraining the uncertainties in the flux to less than one order of magnitude.

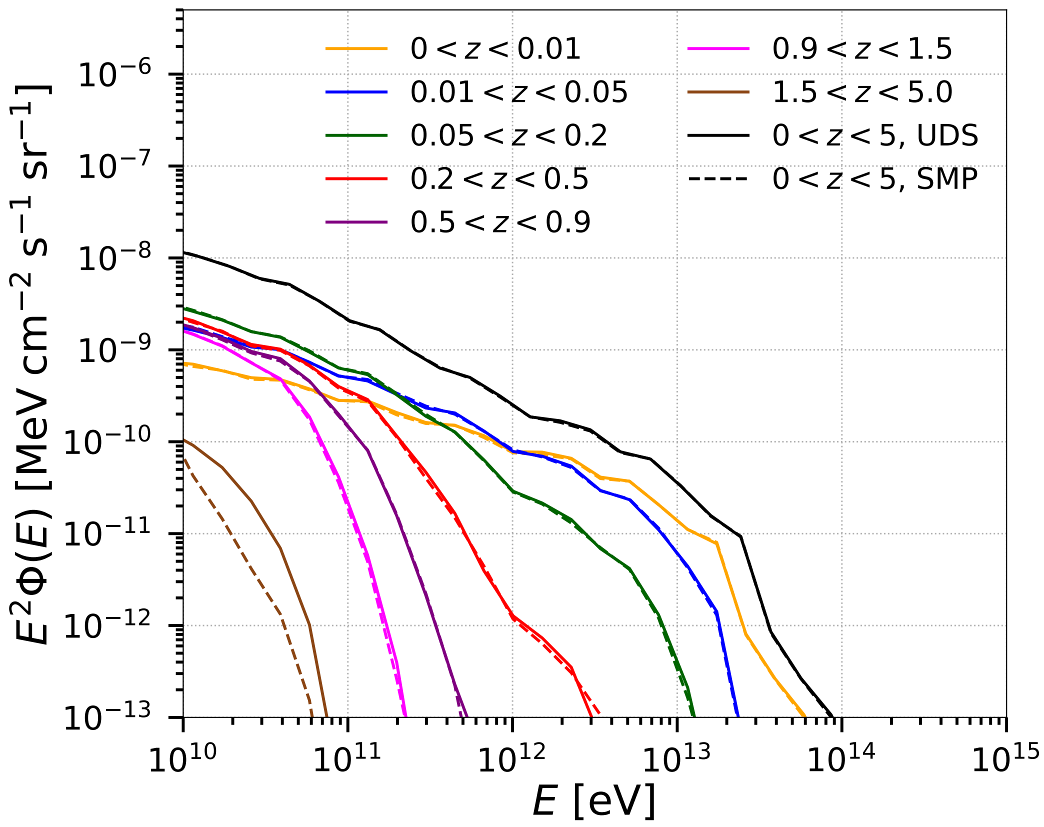

Figure S7. Flux of uniform distribution of CR sources (UDS) vs randomly distributed sources (SMP1), UDS and SMP1 are represented by solid and dashed lines, respectively. The spectral index and cutoff energy is $\alpha = 2.3$ and $E_{\max} = 10^{17}$ eV, respectively. This figure is for the EBL model of ref.[14].

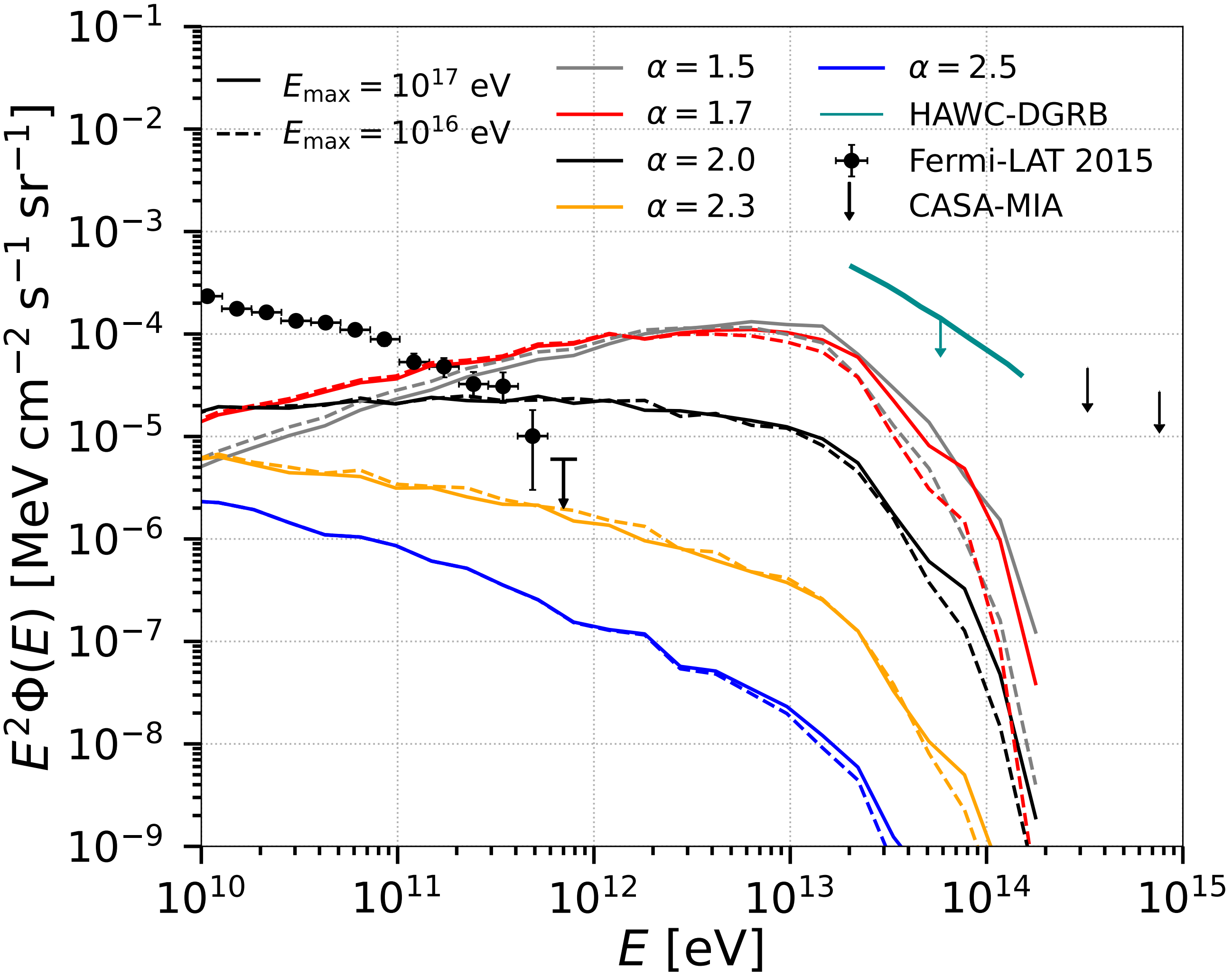

Figure S8. Integrated flux for different combinations of α and $E_{\mathbf{max}}$ and comparison with the Fermi-LAT data (error bars correspond to the total uncertainties, statistical and systematic))[26], and CASA-MIA (90% confidence level)[28] and HAWC DGRB (95% confidence level) upper limits[27].

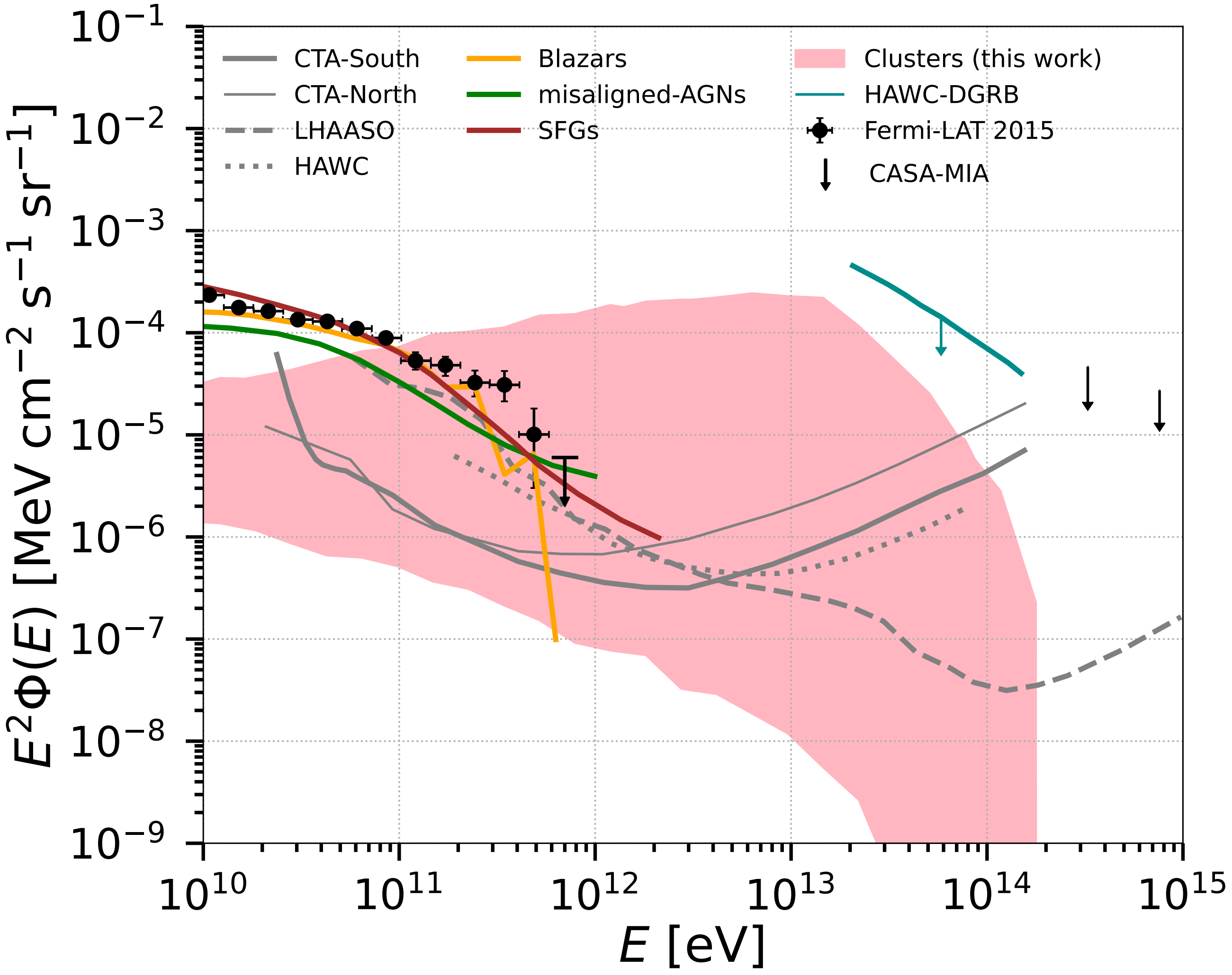

Figure S9. Contribution to DGRB from different types of astrophysical sources. The pink band is plotted for the fiducial range of parameters in our work $\alpha = 1.5 - 2.5$, $E_{\max} = 10^{16} - 10^{17}$ eV. Besides showing the observed DGRB flux from Fermi-LAT (error bars correspond to the total uncertainties, statistical and systematic)[26] and upper limits from HAWC (95% confidence level)[27] and CASA-MIA (90% confidence level)[28], this figure also presents the sensitivity curves obtained for point sources from LHAASO[34], HAWC[35], and the forthcoming CTA North and South observatories[36] for comparison (gray curves). These sensitivity curves are shown only for reference and the scaling factor is simply $\sim$ PSF$^2/4\pi$, where PSF is the point spread function. We also show the contribution from individual sources to the DGRB, namely, blazars[31], AGNs[32], and SFGs[33].

References

1. Dolag, K., Grasso, D., Springel, V. & Tkachev, I. Constrained simulations of the magnetic field in the local universe and the propagation of ultrahigh energy cosmic rays. *J. Cosmol. Astropart. Phys.* **0501**, 009 (2005).
2. Springel, V., Yoshida, N. & White, S. D. Gadget: a code for collisionless and gasdynamical cosmological simulations. *New Astron.* **6**, 79–117 (2001).
3. Springel, V. The cosmological simulation code gadget-2. *Mon. Not. R. Astron. society* **364**, 1105–1134 (2005).
4. Jenkins, A. *et al.* The mass function of dark matter haloes. *Mon. Not. R. Astron. Soc.* **321**, 372–384 (2001).
5. Rosati, P., Borgani, S. & Norman, C. The evolution of x-ray clusters of galaxies. *Annu. Rev. Astron. Astrophys.* **40**, 539–577 (2002).
6. Bocquet, S., Saro, A., Dolag, K. & Mohr, J. J. Halo mass function: baryon impact, fitting formulae, and implications for cluster cosmology. *Mon. Not. R. Astron. Soc.* **456**, 2361–2373 (2016).
7. Giovannini, G., Tordi, M. & Feretti, L. Radio halo and relic candidates from the nrao vla sky survey. *New Astron.* **4**, 141–155 (1999).
8. Tinker, J. *et al.* Toward a halo mass function for precision cosmology: the limits of universality. *Astrophys. J.* **688**, 709 (2008).
9. Alves Batista, R. *et al.* Crpropa 3—a public astrophysical simulation framework for propagating extraterrestrial ultra-high energy particles. *J. Cosmol. Astropart. Phys.* **1605**, 038 (2016).
10. Alves Batista, R. *et al.* CRPropa 3.2 - an advanced framework for high-energy particle propagation in extragalactic and galactic spaces. *J. Cosmol. Astropraticle Phys.* **2022**, 035 (2022).
11. Rodríguez-Ramírez, J. C., de Gouveia Dal Pino, E. M. & Alves Batista, R. VHE Emission from Magnetic Reconnection in the Radiative-Inefficient Accretion Flow of SgrA*. *Astrophys. J.* **879**, 6 (2019). 1904.05765.
12. Kafexhiu, E., Aharonian, F., Taylor, A. M. & Vila, G. S. Parametrization of gamma-ray production cross sections for p p interactions in a broad proton energy range from the kinematic threshold to pev energies. *Phys. Rev. D* **90**, 123014 (2014).
13. Dominguez, A. *et al.* Extragalactic background light inferred from aegis galaxy-sed-type fractions. *Mon. Not. R. Astron. Soc.* **410**, 2556–2578 (2011).
14. Gilmore, R. C., Somerville, R. S., Primack, J. R. & Domínguez, A. Semi-analytic modelling of the extragalactic background light and consequences for extragalactic gamma-ray spectra. *Mon. Not. R. Astron. Soc.* **422**, 3189–3207 (2012).
15. Stecker, F. W., Scully, S. T. & Malkan, M. A. An empirical determination of the intergalactic background light from uv to fir wavelengths using fir deep galaxy surveys and the gamma-ray opacity of the universe. *Astrophys. J.* **827**, 6 (2016).
16. Inoue, S., Sigl, G., Miniati, F. & Armengaud, E. Ultrahigh energy cosmic rays as heavy nuclei from cluster accretion shocks. *arXiv preprint astro-ph/0701167* (2007).
17. Alves Batista, R., de Gouveia Dal Pino, E., Dolag, K. & Hussain, S. Cosmic-ray propagation in the turbulent intergalactic medium. *arXiv preprint arXiv:1811.03062* (2018).

18. Fang, K. & Olinto, A. V. High-energy neutrinos from sources in clusters of galaxies. *Astrophys. J.* **828**, 37 (2016).

19. Heinze, J., Boncioli, D., Bustamante, M. & Winter, W. Cosmogenic neutrinos challenge the cosmic-ray proton dip model. *Astrophys. J.* **825**, 122 (2016).

20. Alves Batista, R., de Almeida, R. M., Lago, B. & Kotera, K. Cosmogenic photon and neutrino fluxes in the auger era. *J. Cosmol. Astropart. Phys.* **1901**, 002 (2019).

21. Lovisari, L., Reiprich, T. & Schellenberger, G. Scaling properties of a complete x-ray selected galaxy group sample. *Astron. & Astrophys.* **573**, A118 (2015).

22. Kotera, K. *et al.* Propagation of ultrahigh energy nuclei in clusters of galaxies: resulting composition and secondary emissions. *Astrophys. J.* **707**, 370 (2009).

23. Ackermann, M. *et al.* Search for cosmic-ray-induced gamma-ray emission in galaxy clusters. *Astrophys. J.* **787**, 18 (2014).

24. Ahnen, M. L. *et al.* Deep observation of the ngc 1275 region with magic: search of diffuse γ-ray emission from cosmic rays in the perseus cluster. *Astron. & Astrophys.* **589**, A33 (2016).

25. Ackermann, M. *et al.* Search for gamma-ray emission from the coma cluster with six years of fermi-lat data. *Astrophys. J.* **819**, 149 (2016).

26. Ackermann, M. *et al.* The spectrum of isotropic diffuse gamma-ray emission between 100 mev and 820 gev. *Astrophys. J.* **799**, 86 (2015).

27. Harding, J. P. Constraints on the diffuse gamma-ray background with hawc. *arXiv preprint arXiv:1908.11485* (2019).

28. Chantell, M. *et al.* Limits on the isotropic diffuse flux of ultrahigh energy γ radiation. *Phys. Rev. Lett.* **79**, 1805 (1997).

29. Pinzke, A. & Pfrommer, C. Simulating the γ-ray emission from galaxy clusters: a universal cosmic ray spectrum and spatial distribution. *Mon. Not. R. Astron. Soc.* **409**, 449–480 (2010).

30. Ackermann, M. *et al.* Resolving the extragalactic γ-ray background above 50 gev with the fermi large area telescope. *Phys. Rev. Lett.* **116**, 151105 (2016).

31. Ajello, M. *et al.* The origin of the extragalactic gamma-ray background and implications for dark matter annihilation. *Astrophys. J. Lett.* **800**, L27 (2015).

32. Di Mauro, M., Calore, F., Donato, F., Ajello, M. & Latronico, L. Diffuse γ-ray emission from misaligned active galactic nuclei. *Astrophys. J.* **780**, 161 (2013).

33. Roth, M. A., Krumholz, M. R., Crocker, R. M. & Celli, S. The diffuse γ-ray background is dominated by star-forming galaxies. *Nature* **597**, 341–344 (2021).

34. Di Sciascio, G., Collaboration, L. *et al.* The lhaaso experiment: from gamma-ray astronomy to cosmic rays. *Nucl. Part. Phys. Proc.* **279**, 166–173 (2016).

35. Abeysekara, A. *et al.* Sensitivity of the high altitude water cherenkov detector to sources of multi-tev gamma rays. *Astropart. Phys.* **50**, 26–32 (2013).

36. CTA Consortium *et al.* *Science with the Cherenkov Telescope Array* (World Scientific, 2018).
